# Dietary oregano essential oil and sodium butyrate enhance growth, immunity, and gene expression in nile tilapia post-*Aeromonas hydrophila* infection

**DOI:** 10.1038/s41598-025-22439-8

**Published:** 2025-11-04

**Authors:** Eman M. Moustafa, Mustafa Shukry, Mona Assas, Haguer M. Salah El Din, Mohamed A. Khallaf, Hanan A. Ghetas, Azza Hafez, Foad Farrag, Asmaa T. Mousa, Wesam H. Marzouk

**Affiliations:** 1 Fish Diseases and Management Department, Faculty of Veterinary Medicine, Faculty of veterinary medicine, Kafrelsheikh University, Kafrelsheikh, Egypt; 2 Animal Physiology Department, Faculty of Veterinary Medicine, Kafrelsheikh University, Kafrelsheikh, Egypt; 3 Fish processing and Biotechnology department, Faculty of Aquatic Fishes, Kafrelsheikh University, Kafrelsheikh, Egypt; 4 Aquatic Animals Medicine and Management Department, Faculty of Veterinary Medicine, Sadat University, Sadat City, 32897 Egypt; 5 Nutrition and Clinical Nutrition Department, Faculty of Veterinary Medicine, Kafrelsheikh University, Kafrelsheikh, Egypt; 6 Anatomy and embryology department, Faculty of Veterinary Medicine, Kafrelsheikh University, Kafrelsheikh, Egypt; 7 Veterinarian at the General Organization for Veterinary Services in Cairo, Cairo, Egypt; 8 Department of Biomedical Sciences, College of Veterinary Medicine, King Faisal University, Al-Ahsa, 31982 Saudi Arabia

**Keywords:** Oregano essential oil, Sodium butyrate, Nile tilapia, Growth performance, Immunity enhancement, Antioxidative response, Aeromonas hydrophila, Animal physiology, Physiology

## Abstract

The impacts of oregano essential oil and sodium butyrate on the growth performance, hematological, biochemical, immunological, antioxidative, and gene expression profiles of Nile tilapia (*Oreochromis niloticus*) fingerlings were examined in this study. One hundred eighty healthy fingerlings (16.00 ± 2.00 g) were acclimated and accidentally divided into three groups, each further split into three replicates. The groups were fed a basal diet (control) or diets supplemented with 0.5% and 1% oregano essential oil and sodium butyrate for eight weeks (The feed additives were administered continuously throughout an 8-week trial period, simulating a practical feeding strategy that would be feasible during high-risk periods (e.g., post-handling stress, seasonal disease outbreaks). Fish fed the supplemented diets demonstrated significantly greater growth performance, with the oregano 1% + sodium butyrate 1% group achieving the highest final body weight (36.91 ± 0.66 g) and lowest feed conversion ratio (1.32 ± 0.04). Hematological indices, including RBC count, Hb, and PCV, were substantially improved. Biochemical analysis revealed reduced ALT, AST, urea, and creatinine levels alongside elevated total protein and globulin concentrations in treated groups. Immunological and antioxidative parameters, such as WBC count, phagocytic activity, SOD, and GPX, showed significant enhancements, particularly in the oregano 1% + sodium butyrate 1% group. Gene expression analysis exposed upregulation of immune-related (TNF-α, IL-1β) and antioxidative (SOD, GPX) genes in supplemented groups, highlighting their role in enhancing health and stress responses. Post *Aeromonas hydrophila* infection, the supplemented groups exhibited improved immunity, antioxidative status, and survival. These findings suggest that dietary supplementation with oregano essential oil and sodium butyrate, particularly at 1% inclusion, effectively enhances growth, health status, immunity, and gene expression in Nile tilapia.

**Supplementary Information:**

The online version contains supplementary material available at 10.1038/s41598-025-22439-8.

## Introduction

The aquaculture industry is having a moment, providing over half the fish consumed worldwide. As the global population increases, the demand for affordable, nutritious fish rises, making aquaculture essential to the global food system. However, challenges such as fish health management, disease control, and environmental impact accompany the industry’s expansion^1^. Nile tilapia, a species extensively cultivated for its flesh, is known for its fast growth, adaptability, and market value. With increasing demand, aquaculture practices must evolve to address these challenges sustainably^2^. A noteworthy issue in aquaculture is the widespread application of antibiotics, leading to antibiotic resistance, where the environment, human health, and fish health are all jeopardized^3^. Overuse of antibiotics can result in resistant pathogens that spread to humans by consuming contaminated fish or water. Additionally, antibiotic residues in effluents can harm aquatic ecosystems, exacerbating the problem of resistance^4^. In response, there is growing interest in natural alternatives like oregano oil and sodium butyrate, which enhance fish health, support immune function, and reduce reliance on antibiotics. Incorporating these functional feed additives into diets can improve sustainability, foster fish welfare, and reduce the environmental effects of antibiotic use^5^. Using functional feed additives in aquaculture offers multiple advantages, enhancing fish health, productivity, and sustainability^6^. Additives like oregano oil and sodium butyrate improve immune function, digestion, and gut health, boosting growth and disease resistance in Nile tilapia^2^. These natural alternatives reduce the reliance on synthetic chemicals and antibiotics, ensuring food safety and environmental sustainability^3^. Originating from the plant Origanum vulgare, oregano essential oil (OEO) is known for its ability to fight against microbes, antioxidants, and inflammation^7^. It offers a promising solution to bacterial infections in aquaculture^8^. Research shows OEO enhances immune responses, reduces oxidative stress, and inhibits harmful microorganisms in tilapia^9^. Additionally, it improves growth performance and feed efficiency, promoting fish health without synthetic chemicals^10^. Aquaculture can reduce antibiotic use by incorporating oregano oil, supporting a more sustainable system^8^. This is especially crucial in the context of rising antibiotic resistance, a grave danger to ecosystems and people alike^11^. Thus, oregano oil is valuable for minimizing pharmaceutical use and enhancing aquaculture sustainability^12^.

Nile tilapia (*Oreochromis niloticus*) is among the most economically important freshwater fish species globally, ranked as the second most farmed fish after carp^13^. It is widely cultivated due to its fast growth, adaptability to various farming systems, omnivorous feeding habits, and high market demand^14^. According to the Food and Agriculture Organization (FAO), global tilapia production exceeded 6.4 million metric tons in 2022, with an estimated market value surpassing USD 11 billion, highlighting its vital role in worldwide aquaculture and food security^15^. Nile tilapia is the cornerstone of the aquaculture sector in Egypt, accounting for approximately 65–70% of the country’s total farmed fish production. Egypt is the top producer of tilapia in Africa and ranks among the top three producers worldwide, with an annual production exceeding 1.6 million tons^16^. The species supports thousands of small-scale farmers and contributes significantly to rural livelihoods, national protein intake, and export potential, making its health and sustainability a national priority.

Sodium butyrate is classified as a nature-identical compound, the salt form of butyric acid, a short-chain fatty acid (SCFA) naturally produced through microbial fermentation in the intestines. Although synthetically manufactured to ensure stability and precise dosing, sodium butyrate mimics endogenously produced metabolites and is widely utilized as a functional feed additive in aquaculture. It has demonstrated notable potential in supporting gut health by strengthening the intestinal mucosal barrier and enhancing the proliferation of beneficial gut microbiota^17^. In Nile tilapia, sodium butyrate improves digestion, enhances nutrient absorption, and bolsters immune function. Fostering a healthy gut and balanced microbiota contributes to better growth, disease resistance, and overall fish well-being^17^. Additionally, sodium butyrate helps reduce inflammation, particularly in the digestive system and alleviates stress, which is common in aquaculture^18^. Including sodium butyrate in tilapia diets aligns with the growing demand for natural, sustainable aquaculture solutions. It enhances fish health and productivity while minimizing environmental impact^18^. Furthermore, functional feed additives help maintain aquatic balance by reducing harmful substances in the water, leading to a cleaner, more sustainable aquaculture system that benefits both fish and the ecosystem^19^. As the industry faces ongoing challenges related to disease management and environmental sustainability, using functional additives like oregano oil and sodium butyrate will be pivotal in determining aquaculture’s trajectory^20^. The individual benefits of oregano essential oil (OEO) and sodium butyrate are well documented: in tilapia and other fishes, OEO improves growth and feed efficiency and enhances innate immunity and antioxidative status^9,10,12^; sodium butyrate strengthens intestinal integrity and histomorphometry, elevates digestive and antioxidant enzyme activities, and upregulates immune-related genes in Nile tilapia^17,18,21^ However, evidence evaluating their combined use in finfish diets remains comparatively scarce, and equi-ratio co-supplementation (e.g., 0.5%+0.5% or 1%+1%) has rarely been tested. Moreover, responses to each additive are generally beneficial but not uniformly consistent across studies, varying with dose, formulation (protected vs. unprotected), diet, life stage, and health/challenge status^18,22^. Combining OEO and sodium butyrate may offer additive or synergistic effects by targeting systemic immunity and intestinal health, which are essential for disease resilience and performance in aquaculture. While their individual benefits are documented, evidence of their combined use remains limited, particularly in equal ratios. study aims to fill that gap by evaluating whether their integration confers superior protection and physiological benefits in Nile tilapia subjected to *Aeromonas hydrophila* challenge. This research investigated the effects of incorporating oregano essential oil and sodium butyrate into Nile tilapia diets on growth performance, physiological responses, oxidative balance, and immune function when subjected to *Aeromonas hydrophila* infection.

## Materials and methods

### Animal ethics

All experimental procedures were performed in compliance with applicable guidelines and ethical standards. Qualified researchers conducted fish handling and all related protocols following the ethical guidelines set by the Animal Ethics Committee of Kafrelsheikh University, Egypt (KFS-IACUC/137/2023), which oversees animal welfare and research practices. The study adhered to the standards and recommendations outlined in the ARRIVE (Animals in Research: Reporting In Vivo Experiments) guidelines^23^.

### Preparation of the experimental diet

A fundamental diet was developed by incorporating commercial products, such as fish meal, soybean meal, corn gluten meal, yellow corn grains, wheat bran, minerals mix, vitamins, and fish oil (Table S1). The dry materials were ground into fine particles using a feed processor. Three diets were developed by supplementing basal diets with oregano essential oil (Oregano essential oil was provided by ELHAWAG Company (Giza, Egypt; carvacrol: 60.2% and thymol: 2%) and sodium butyrate at varying concentrations (0.5, 1 gm/kg ration). The contents were weighed and combined for twenty minutes using a combination blender. After mixing the ingredients with water, oil, dicalcium phosphate, vitamins, and minerals, the dough was extruded through a 2 to 3 mm die using a pelleting machine. Once the granules were air-dried, they were placed in a refrigerator and kept at 4 °C. Using an established procedure, we confirmed that the test diets had the same nutritional profile^24^.

### Experimental design

A total of 180 healthy Nile tilapia (*Oreochromis niloticus*) fingerlings (average initial weight: 16.00 ± 2.00 g) were obtained from a private fish farm in Kafrelsheikh, Egypt. Kafrelsheikh University’s Faculty of Veterinary Medicine’s Department of Fish Diseases and Management received the fingerlings. The fish were placed in fiberglass tanks as soon as they arrived and given a basal diet with about 30% crude protein (CP) for two weeks to help them adjust.

Following acclimation, the fish were randomly allocated into three groups of 60 individuals, with each group further divided into three replicates of 20 fish. They were maintained in glass aquariums (60 × 30 × 40 cm, 70 L capacity) equipped with efficient oxygenation systems. The first group (control) was provided with a commercial basal diet. The commercial basal diet was formulated to contain approximately 30% crude protein, 6% lipid, 12% moisture, and 8% ash, aligning with the nutritional requirements for Nile tilapia fingerlings. Ingredients included fish meal, soybean meal, corn gluten meal, yellow corn, wheat bran, fish oil, a vitamin–mineral premix, and dicalcium phosphate. The dry ingredients were ground and thoroughly mixed for 20 min using a horizontal mixer. After adding oil and water to form a homogenous dough, pellets were extruded using a 2–3 mm die, air-dried at room temperature, and stored at 4 °C until use.

In contrast, the second and third groups were fed diets supplemented with 0.5 g/kg and 1 g/kg of oregano essential oil and sodium butyrate (AVITASA, Spain) was used as a nature-identical feed additive, respectively. For eight weeks, the fish were fed experimental diets at a rate of 3% of the total aquarium biomass, with feed amounts adjusted every two weeks based on weight measurements. Waste and uneaten feed were removed daily through siphoning, and 50% of the aquarium water was replaced with dechlorinated freshwater. A Lamotte water analysis kit (USA) monitored water quality parameters biweekly.

The inclusion levels were selected within ranges reported as effective in aquaculture. For oregano essential oil (OEO), prior work in tilapia and other teleosts shows improvements in growth, feed efficiency, innate immunity, and antioxidative status at ~ 0.3–1.0 g/kg diet^9,10,12,25,44,53^. For sodium butyrate, studies in Nile tilapia report benefits to intestinal histomorphometry, digestive and antioxidant enzymes, and immune-related gene expression across ~ 0.5–2.0 g/kg^17,18,21^. We therefore evaluated 0.5 and 1.0 g/kg for each additive, and used equal-ratio co-supplementation.

During the study, water temperature ranged from 24 to 27 °C, dissolved oxygen levels averaged 6.5 ± 0.5 mg/L, pH was recorded at 7.1 ± 0.8, and electrical conductivity (EC) was measured at 219 ± 2 µmho/cm. Ammonia levels were maintained below 0.1 mg/L, and a 12:12 h light-dark photoperiod was observed. Water quality assessments adhered to the guidelines established by^26^ standards.

### Measurements of fish growth

Twenty fish per replication were precisely measured after 8 weeks using an electronic balance. The methodology described by^2^ was employed to ascertain all growth parameters, including the feed conversion ratio (FCR), average daily gain (ADG), specific growth rate (SGR), survival rate (SR%), and total weight gain (TWG).

- The following standard formulas were used to calculate growth and feed utilization indices:
- Weight gain (WG, g) = Final body weight (FBW) – Initial body weight (IBW).
- Specific growth rate (SGR, %/day) = [(ln FBW – ln IBW)/Number of days] × 100.
- Feed conversion ratio (FCR) = Total feed intake (g)/Weight gain (g).
- Average daily gain (ADG, g/day) = Weight gain/Number of days.
- Survival rate (%) = (Final number of fish/Initial number of fish) × 100.

### Biochemical and hematological parameters

The fish were starved for 24 h and sedated with 100 mg/L tricaine methane-sulfonate before sampling^27^. Each group randomly chose nine fish, with three fish per replication. After weighing each fish, blood was extracted from its caudal vessels using 5 mL syringes. Blood samples were segregated into two parts: one for immediate hematological analysis and leukocyte counts in EDTA-heparinized containers and the other for biochemical tests. Hematological parameters, including RBC and WBC counts, hemoglobin concentration, PCV%, MCHC, and MCV, were measured using standard methods^28^. Differential WBC counts followed^29^, as well as biochemical parameters. Using colorimetric techniques, the quantities of total protein (TP) and albumin (Alb) were assessed after^30,31^, respectively, while globulin (Glo) was computed by subtracting albumin from total protein. Liver enzymes (AST and ALT) were measured colorimetrically following^32^. Serum creatinine and urea levels were verified colorimetrically following^33,34^.

### Determination of immune parameters

Phagocytic activity and phagocytic index were assessed following^35^. Lysozyme activity was measured following^36^using the lysis of Gram-positive bacteria, *Micrococcus lysodeikticus* (Sigma, St. Louis, MO). Glucose (GOD-PAP) (VitroScient, Egypt) and cholesterol (Biodiagnostic, Egypt) levels were determined colorimetrically at 500 nm based on^37,38^, respectively. Triglycerides (Biodiagnostic, Egypt) were assessed at 505 nm following^39^. Lipase (Spectrum, Egypt) and amylase (Biodiagnostic, Egypt) were determined at 580 nm and 660 nm following^40,41^, respectively. Serum levels of superoxide dismutase (SOD), catalase (CAT), glutathione peroxidase (GPx), and malondialdehyde (MDA) were measured using commercial diagnostic kits (Cusabio Biotech Co., Ltd., China), following the manufacturer’s instructions. Blood samples were centrifuged at 3,000 rpm for 15 min at 4 °C to obtain serum. For SOD (CSB-E15929Fh), CAT (CSB-E15928Fh), and GPx (CSB-E15930Fh) assays, reagents and samples were prepared as directed, and wells were set with appropriate blanks. Subsequently, 50 µL of either standard or serum sample was added to each well, followed by 50 µL of HRP conjugate (1X) to all wells except the blanks. The plates were incubated at 37 °C for 1 h, washed five times, and then 90 µL of TMB substrate was added. After incubation in the dark at 37 °C for 30 min, 50 µL of stop solution was added, and absorbance was read at 450 nm within 10 min. For MDA determination, the method of Ledwozyw et al.^42^was followed: 1 mL of serum was mixed with 2 mL of trichloroacetic acid and hydrochloric acid under acidic conditions, diluted to 200 mL with distilled water, incubated for 30 min, cooled, and centrifuged at 300 rpm for 10 min. The supernatant was then collected, and absorbance was measured at 535 nm.

### The challenge with *Aeromonas hydrophila*

The experimental infection was carried out through an intraperitoneal injection of 0.2 ml of *Aeromonas hydrophila* culture at a concentration of 3 × 10⁷ CFU following^43^. Mortalities were recorded daily for 15 days post-infection. *The drop plate procedure* determined the *A. hydrophila* concentration and injection dose. The strain was cultured on Tryptic Soy Agar (TSA) for 24 h at 37 °C with constant shaking (250 rpm), suspended in sterile saline, and diluted (10² to 10⁷ CFU). Fish were injected with 0.2 ml of each dilution and observed for two weeks. Mortalities were recorded twice daily, and deceased fish were examined. The lethal dose (LD50) was calculated as the dose causing 50% mortality following^44^.

### Gene expression analysis

Following the manufacturer’s instructions, RNA was extracted from 50 mg of *Oreochromis niloticus* spleen tissue using Trizol reagent (iNtRON Biotechnology). Integrity was verified on a 2% agarose gel, and purity and concentration were measured with a Nanodrop BioDrop spectrophotometer (Biochrom Ltd, UK). Two micrograms of RNA were reverse transcribed into cDNA using the ABT 2X RT Mix kit. Gene expression of inflammatory (Nf-κb, TNF-α, IL-1β) and antioxidant (CAT, GPx) genes was analyzed using a Rotor-Gene Q system (Qiagen, Germany). qPCR was performed in a 20 µL reaction with SYBR Green, primers, cDNA, and nuclease-free water. The protocol involved denaturation at 95 °C for 15 min, 40 cycles at 95 °C for 10 s, annealing for 15 s, and extension at 72 °C for 25 s, followed by melt curve analysis. Relative expression was calculated using the 2 − ΔΔCT method^45^, normalized against β-actin and EF1A.

### Histopathological examinations (morphometry of intestinal villi)

Five fish were dissected for histomorphometric evaluation after being anesthetized with 40% ethyl alcohol in each of the three groups: control, 0.5 g/kg oregano essential oil, and 1 g/kg oregano essential oil. After dehydrating in graded ethanol, cleaning in xylene, and embedding in paraffin, samples of the anterior intestine were fixed in Bouin’s solution. The sections were stained with hematoxylin and eosin at a thickness of 4–5 μm^46^. Imaging software (NIH, Bethesda, MD) measured intestinal villi length, width, crypt depth, and length/crypt depth ratio. Averages (± SE) were derived from ten villi and crypts from five cross-sections per fish.

### Data analysis

Data are presented as mean ± SEM. One-way ANOVA was applied, and post hoc comparisons were conducted using Tukey’s multiple-comparison test (*p* < 0.05). Statistical analyses were performed in GraphPad Prism (version 9.5; GraphPad Software, San Diego, CA, USA).

## Results

### Growth and feed utilization

The growth performance and feed utilization data for Nile tilapia-fed diets with varying doses of oregano and sodium butyrate are presented in Table 1. Fish in the supplemented groups showed significantly improved growth parameters compared to the control group (*p* < 0.05). The group fed a diet containing 1% oregano and 1% sodium butyrate achieved the highest final body weight (FBW) of 36.91 ± 0.66 g and weight gain (WG) of 21.58 ± 0.76 g. Conversely, the control group exhibited the lowest WG (15.58 ± 0.55 g) and FBW (32.42 ± 0.98 g). Total feed intake was lower in the supplemented groups, while the control group consumed the most feed (35.90 ± 0.67 g). Feed conversion ratio (FCR) was markedly better in the groups receiving supplementation, with the lowest FCR of 1.32 ± 0.04 observed in the group fed 1% oregano and 1% sodium butyrate. Specific growth rate (SGR) and average daily gain (ADG) were also substantially superior in the supplemented groups compared to the control, indicating the beneficial outcomes of dietary oregano and sodium butyrate on growth efficiency.

**Table 1:** Nile tilapia’s growth and feed utilization parameters (*Oreochromis niloticus*) fed on doses of oregano and sodium butyrate for 56 days.

Parameters	Control	Oregano 0.5 + sod 0.5	Oregano1 + sod 1	*p*. value
IBW (g)	15.83$\:\pm\:0.44$	15.50$\:\pm\:0.29$	15.33$\:\pm\:0.17$	0.5615
FBW (g)	32.42$\:\pm\:0.98$^b^	36.38$\:\pm\:0.76$^a^	36.91$\:\pm\:0.66$^a^	0.0151
WG (g)	15.58$\:\pm\:0.55$^b^	20.88$\:\pm\:0.69$^a^	21.58$\:\pm\:0.76$^a^	0.0014
Total feed intake(g)	35.90$\:\pm\:0.67$^a^	30.53$\:\pm\:0.74$^b^	28.33$\:\pm\:0.15$^b^	0.0002
FCR	2.31$\:\pm\:0.05$^a^	1.46$\:\pm\:0.01$^b^	1.32$\:\pm\:0.04$^b^	< 0.0001
SGR (% day^−1^)	0.28$\:\pm\:0.01$^b^	0.37$\:\pm\:0.01$^a^	0.38$\:\pm\:0.01$^a^	0.0013
ADG	27.83$\:\pm\:0.98$^b^	37.28$\:\pm\:1.23$^a^	38.50$\:\pm\:1.39$^a^	0.0015

IBW; Initial body weight, FBW; Final body weight, WG; Weight gain, FCR; Feed conversion ratio, SGR; Specific growth rate, ADG; Average daily gain. Data are expressed as Mean ± SEM where *n* = 3 as triplicate tanks. Values with different superscripts within a row significantly differ (*p* < 0.05).

### Hematological parameters

Table 2 showed that the hematological analysis showed that RBC, Hb levels, and PCV were extensively elevated in the oregano and sodium butyrate-supplemented groups compared to the control. The highest RBC count (4.52 ± 0.28 × 10⁶/mm³), Hb (13.00 ± 0.91 g/100 ml), and PCV (43.33 ± 2.91%) were observed in the oregano 1% + sodium butyrate 1% group. The control group had the lowest values, with RBCs at 2.83 ± 0.54 × 10⁶/mm³, Hb at 8.35 ± 1.35 g/100 ml, and PCV at 26.67 ± 5.36%. The treatments did not significantly alter other parameters such as MCV, MCH, and MCHC. The WBC and heterophil counts were significantly higher in the supplemented groups, with the oregano 1% + sodium butyrate 1% group showing the highest values (60.50 ± 9.05 × 10³/mm³ and 11.36 ± 2.08 × 10³/mm³, respectively) compared to the control. Lymphocyte and monocyte count also increased significantly, while basophil and eosinophil percentages remained unchanged Table 3.

**Table 2:** Effect of dietary level of oregano and sodium butyrate for 56 days on hematological parameters of nile tilapia (*Oreochromis niloticus*).

Parameters	Control	Oregano 0.5 + sod 0.5	Oregano1 + sod 1	*p*. value
RBCs (x10^6^/mm³)	2.83$\:\pm\:0.54$^b^	4.20$\:\pm\:0.10$^ab^	4.52$\:\pm\:0.28$^a^	0.0332
Hb (g/100 ml)	8.35$\:\pm\:1.35$^b^	12.20$\:\pm\:0.20$^ab^	13$\:\pm\:0.91$^a^	0.0279
PCV (%)	26.67$\:\pm\:5.36$^b^	39.33$\:\pm\:0.67$^ab^	43.33$\:\pm\:2.91$^a^	0.0367
MCV	92.77$\:\pm\:1.07$	93.77$\:\pm\:0.65$	95.93$\:\pm\:1.21$	0.2868
MCH	29.77$\:\pm\:0.79$	29.10$\:\pm\:0.68$	28.73$\:\pm\:0.30$	0.5320
MCHC	31.80$\:\pm\:1.21$	31.07$\:\pm\:0.54$	29.93$\:\pm\:0.09$	0.2929

Mean ± SEM where *n* = 3 as triplicate tanks. Values with different superscripts within a row significantly differ (*p* < 0.05).

**Table 3:** Effect of dietary level of oregano and sodium butyrate for 56 days on the leucocytic profile of nile tilapia (*Oreochromis niloticus*).

Parameters	Control	Oregano 0.5 + sod 0.5	Oregano1 + sod 1	*p*. value
WBCs (x10³/mm³)	17.53$\:\pm\:$2.62^b^	42.30$\:\pm\:$1.48^a^	40.67$\:\pm\:$2.12^a^	0.0003
Heterophil (%)	20.00$\:\pm\:$2.31	17.00$\:\pm\:$1	15.67$\:\pm\:$0.88	0.2062
Lymphocyte (%)	70.33$\:\pm\:$0.33	73.00$\:\pm\:$2	75.00$\:\pm\:$1.15	0.4219
Monocyte (%)	7.33$\:\pm\:$1.45	7.66$\:\pm\:$0.67	7.33$\:\pm\:0.33$	0.9595
Eosinophil (%)	1.33$\:\pm\:$0.33	1.00$\:\pm\:$0	1.00$\:\pm\:$0	0.4219
Basophil (%)	1.00$\:\pm\:$0^ab^	1.67$\:\pm\:$0.33^ab^	1.00$\:\pm\:$0^ab^	0.0787
Heterophil (x10³/mm)	3.62$\:\pm\:$0.95^b^	7.15$\:\pm\:$0.71^a^	6.34$\:\pm\:$0.23^ab^	0.0264
Lymphocyte (x10³/mm	12.23$\:\pm\:$1.44^b^	30.43$\:\pm\:$0.62^a^	30.53$\:\pm\:$1.96^a^	0.0002
Monocyte (x10³/mm³)	1.30$\:\pm\:$0.31^b^	3.22$\:\pm\:$0.42^a^	2.98$\:\pm\:$0.18^a^	0.0101
Eosinophils(x10³/mm³)	0.23$\:\pm\:$0.05^b^	0.41$\:\pm\:$0.01^a^	0.41$\:\pm\:0.02$^a^	0.0182
Basophil(x10³/mm³)	0.18$\:\pm\:$0.02^b^	0.43$\:\pm\:$0.03^a^	0.41$\:\pm\:0.02$^a^	0.0010

Mean ± SEM where *n* = 3 as triplicate tanks. Values with different superscripts within a row significantly differ (*p* < 0.05).

### Biochemical parameters

Table 4 showed that dietary supplements substantially influenced the biochemical parameters of Nile tilapia, including ALT, AST, total protein, albumin, globulin, urea, and creatinine. ALT and AST levels were notably lower in the supplemented groups compared to the control group. The group fed a combination of 1% oregano and 1% sodium butyrate demonstrated the lowest ALT (28.85 ± 1.58 U/L) and AST (25.36 ± 3.14 U/L) levels. Total protein and globulin concentrations were markedly higher in the supplemented groups. The highest levels were in the oregano 1% + sodium butyrate 1% group, with total protein reaching 4.90 ± 0.07 g/dL and globulin 2.93 ± 0.28 g/dL. Furthermore, urea and creatinine levels were significantly reduced in the supplemented groups compared to the control, indicating improved biochemical profiles with dietary supplementation.

**Table 4:** Effect of dietary level of oregano and sodium butyrate for 56 days on biochemical parameters of nile tilapia (*Oreochromis niloticus*).

Parameters	Control	Oregano 0.5 + sod 0.5	Oregano1 + sod 1	*p*. value
ALT (U/l)	36.77$\:\pm\:1.88$^a^	29.25$\:\pm\:1.61$^b^	28.85$\:\pm\:1.58$^b^	0.0275
AST (U/l)	34.47$\:\pm\:3.47$^a^	20.78$\:\pm\:0.22$^b^	25.36$\:\pm\:3.14$^ab^	0.0300
Total Protein (g/dl)	3.80$\:\pm\:0.28$^b^	3.78$\:\pm\:0.05$^a^	4.90$\:\pm\:0.07$^a^	0.0066
Albumin (g/dl)	1.16$\:\pm\:0.07$^b^	1.41$\:\pm\:0.05$^a^	1.34$\:\pm\:0.04$^ab^	0.0449
Globulin (g/dl)	2.12$\:\pm\:0.06$^b^	3.17$\:\pm\:0.23$^a^	2.93$\:\pm\:0.28$^ab^	0.0289
Urea (mg/dl)	3.25$\:\pm\:0.30$^a^	1.77$\:\pm\:0.22$^b^	1.72$\:\pm\:0.27$^b^	0.0101
Creatinine (mg/dl)	1.42$\:\pm\:0.13$^a^	1.10$\:\pm\:0.05$^ab^	1.04$\:\pm\:0.01$^b^	0.0311

ALT = alanine aminotransferase; AST = aspartate aminotransferase. Mean ± SEM where *n* = 3 as triplicate tanks. Values with different superscripts within a row significantly differ (*p* < 0.05).

### Anti-oxidative parameters

**Fig. 1:**
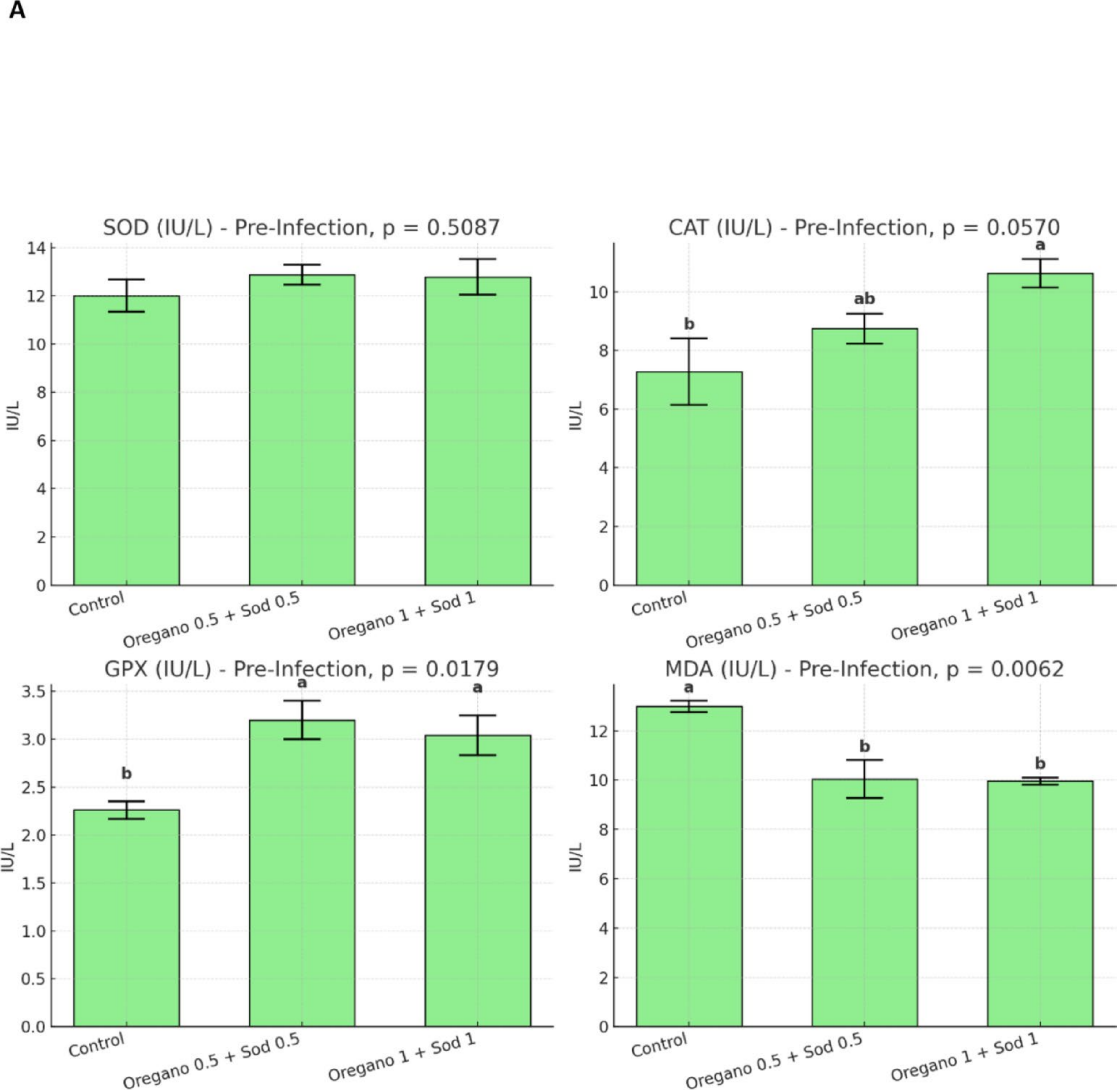

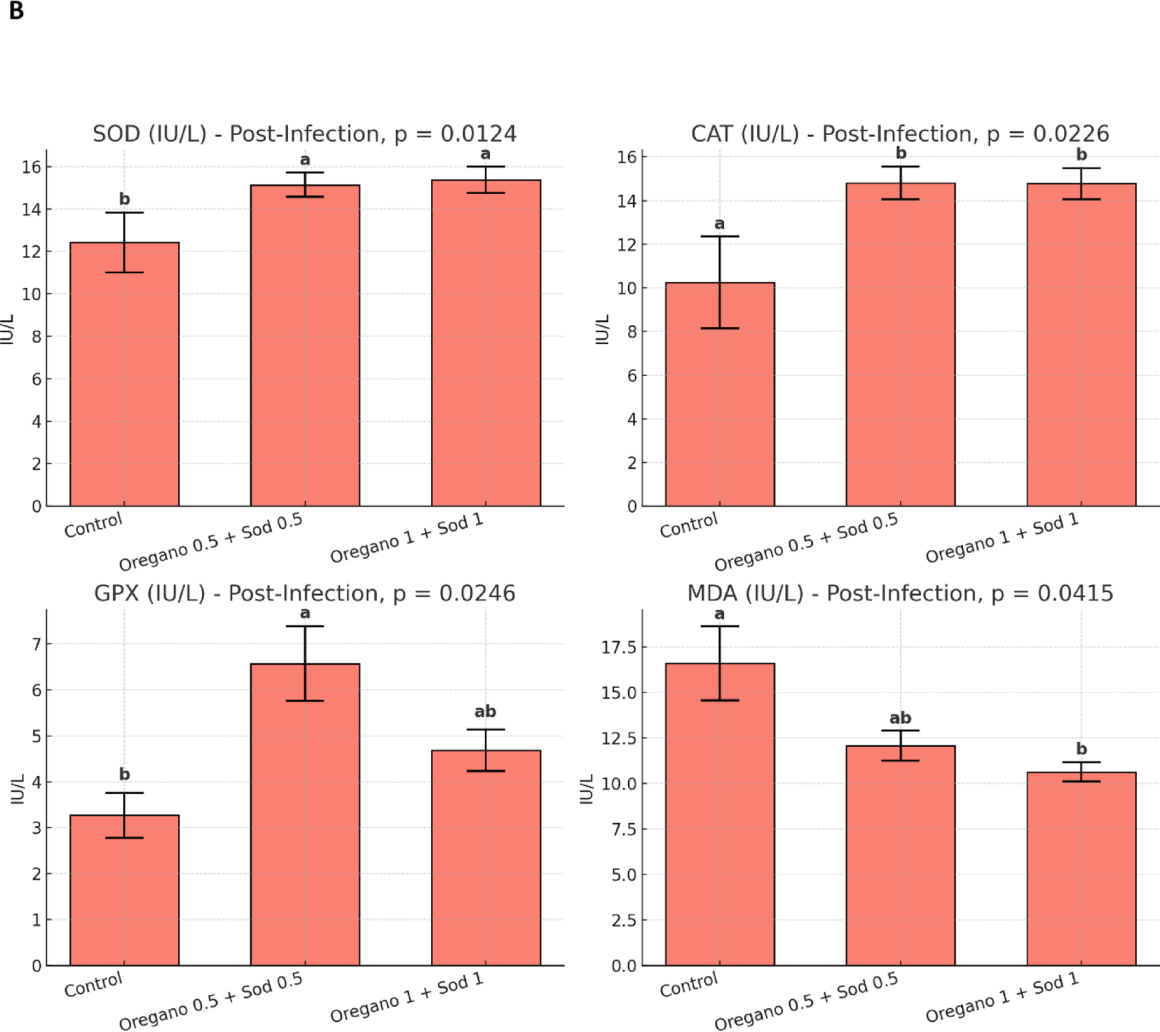
Effect of dietary level of Oregano and sodium butyrate for 56 days on anti-oxidative parameters of Nile tilapia pre- (**A**) and post-*Aeromonas hydrophila* infection (**B**). Mean ± SEM where *n* = 3 as triplicate tanks. Values with different superscripts within a row significantly differ (*p* < 0.05).

Dietary supplementation with oregano essential oil and sodium butyrate significantly modulated antioxidant enzyme activities in Nile tilapia. Before infection, fish receiving the supplemented diets exhibited significantly higher GPX activity (*p* = 0.0179) and lower MDA levels (*p* = 0.0062), indicating enhanced antioxidant capacity and reduced lipid peroxidation. Post-infection, this effect became more pronounced; both SOD (*p* = 0.0124), CAT (*p* = 0.0226), and GPX (*p* = 0.0246) activities were significantly elevated in the supplemented groups compared to control. At the same time, MDA levels were notably reduced (*p* = 0.0415). These findings demonstrate that the combined supplementation of oregano and sodium butyrate, particularly at 0.5% and 1%, enhanced the antioxidative status of tilapia both before and after bacterial challenge, contributing to improved oxidative stress defense. Figure 1.

### Immunological parameters

**Fig. 2:**
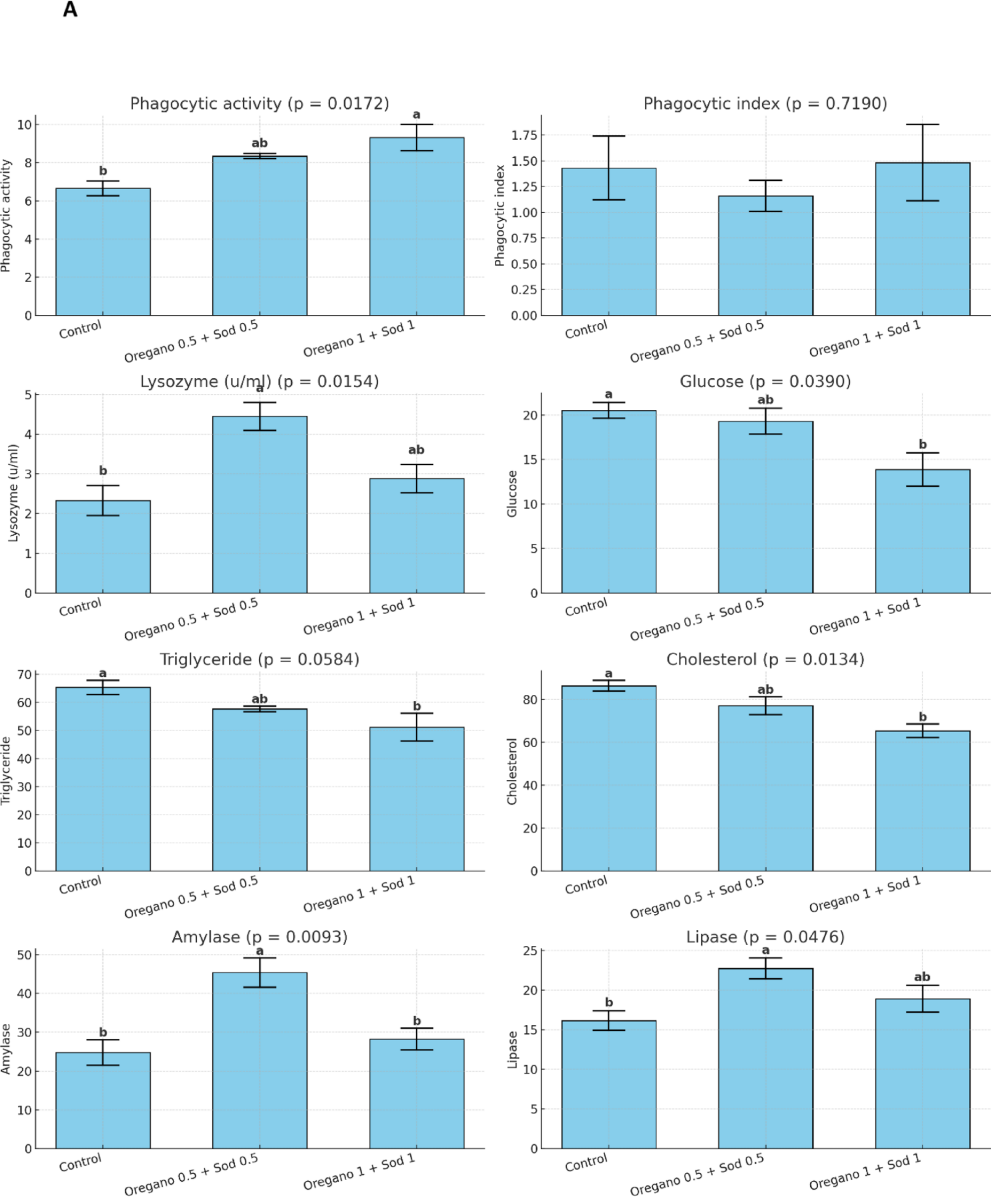

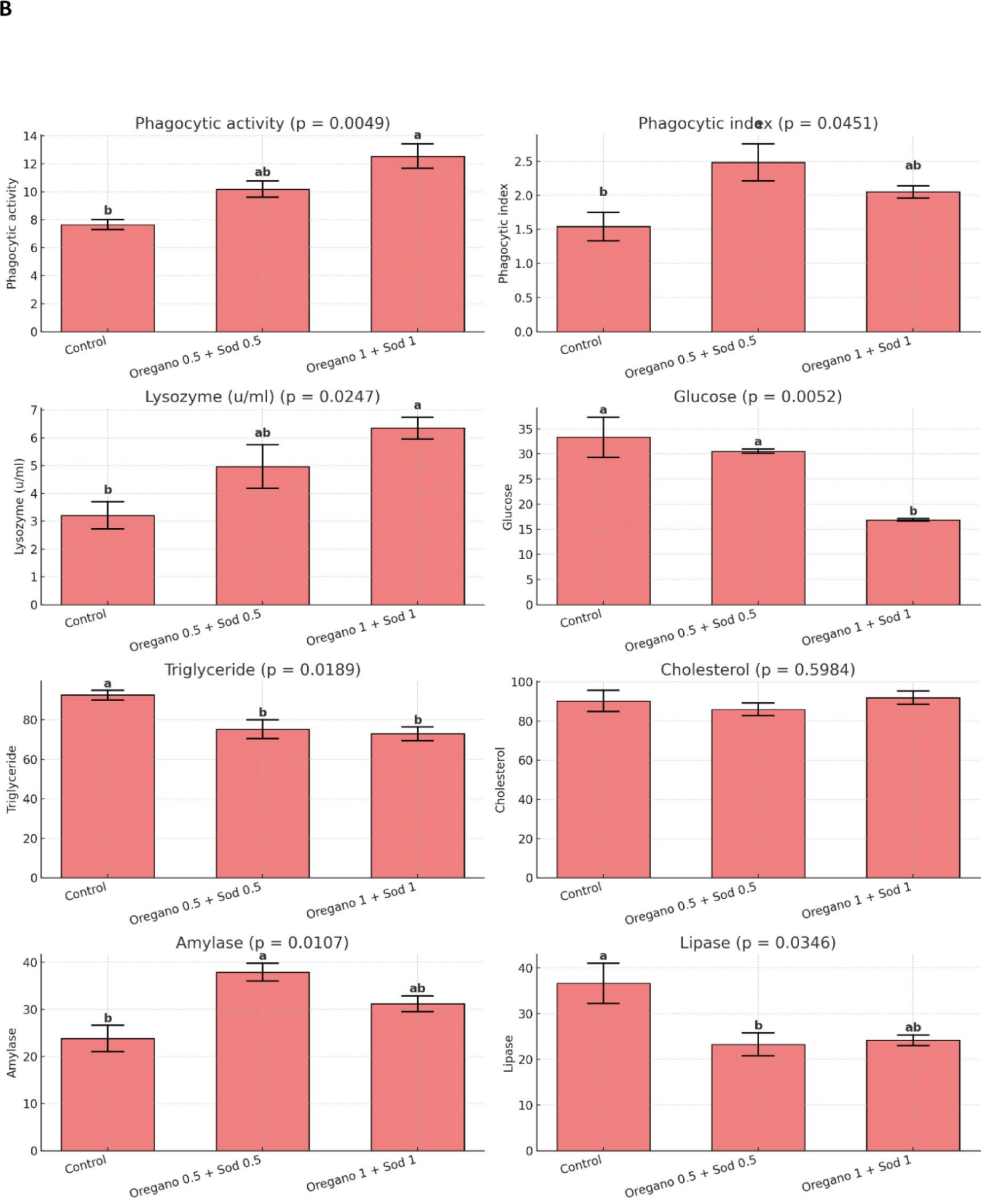
Effect of dietary level of Oregano and sodium butyrate for 56 days on anti-oxidative parameters of Nile tilapia pre and post- *Aeromonas hydrophila* infection. Mean ± SEM where *n* = 3 as triplicate tanks. Values with different superscripts within a row significantly differ (*p* < 0.05).

Phagocytic activity was considerably improved in the supplemented groups, with the oregano 1% + sodium butyrate 1% group showing the highest phagocytic activity (9.31 ± 0.68). Lysozyme activity was also known to be higher in the supplemented groups, with the oregano 0.5% + sodium butyrate 0.5% group showing the highest lysozyme activity (4.45 ± 0.35 u/ml). Glucose, triglyceride, and cholesterol levels were substantially lower in the supplemented groups, particularly in the oregano 1% + sodium butyrate 1% group. Amylase and lipase activities were also significantly improved in the supplemented groups Figure. 2.

### Post *Aeromonas hydrophila* infection

After the post-infection period with *Aeromonas hydrophila*, significant improvements in RBCs, Hb, and PCV were observed in the supplemented groups compared with the control. The highest values for these markers were found in the oregano 1% + sodium butyrate 1% group, with RBCs at 4.50 ± 0.28 × 10⁶/mm³, Hb at 13.30 ± 0.68 g/100 ml, and PCV at 44.00 ± 2.64%. In contrast, the control group showed significantly lower values, as shown in Table 5. Similarly, WBC count, heterophil count, and lymphocyte count were higher in the supplemented groups, particularly in the oregano 1% + sodium butyrate 1% group Table 6. After *Aeromonas hydrophila* infection, dietary supplementation with oregano (0.5% and 1%) and sodium butyrate improved biochemical parameters in Nile tilapia. ALT and AST levels significantly decreased, with the lowest levels in the 1% oregano + 1% sodium butyrate group. Total protein levels increased significantly, especially in the 0.5% oregano + 0.5% sodium butyrate group. At the same time, albumin, globulin, urea, and creatinine showed no significant differences except for a slight increase in creatinine in the highest supplementation group. These results indicate enhanced liver function and protein metabolism with supplementation Table 7. SOD, CAT, GPX, and MDA levels also showed improvements, with the oregano 1% + sodium butyrate 1% group showing the lowest MDA and highest GPX levels Fig. 1. Phagocytic and lysozyme activity were significantly enhanced in the supplemented groups, indicating improved immune response post-infection Figure. 2.

**Table 5:** Effect of dietary level of oregano and sodium butyrate for 56 days on hematological parameters of nile tilapia (*Oreochromis niloticus*) post *Aeromonas hydrophila* infection.

Parameters	Control	Oregano 0.5 + sod 0.5	Oregano1 + sod 1	*p*. value
RBCs (x10^6^/mm³)	2.82$\:\pm\:$0.41^b^	4.10$\:\pm\:$0.13^ab^	4.50$\:\pm\:$0.28^a^	0.0179
Hb (g/100 ml)	7.80$\:\pm\:$1.12^b^	12.40$\:\pm\:$0.29^a^	13.30$\:\pm\:$0.68^a^	0.0051
PCV (%)	27.00$\:\pm\:4.35$^b^	40.33$\:\pm\:$1.76^ab^	44.00$\:\pm\:2.64$^a^	0.0190
MCV	95.33$\:\pm\:$2.02	98.17$\:\pm\:$1.17	97.83$\:\pm\:$0.48	0.3493
MCH	27.70$\:\pm\:$0.35	30.13$\:\pm\:$0.72	29.57$\:\pm\:$0.69	0.0692
MCHC	29.10$\:\pm\:$0.51	30.70$\:\pm\:$1.02	30.27$\:\pm\:$0.72	0.3889

Mean ± SEM where *n* = 3 as triplicate tanks. Values with different superscripts within a row significantly differ (*p* < 0.05).

**Table 6:** Effect of dietary level of oregano and sodium butyrate for 56 days on leucocytic profile of nile tilapia (*Oreochromis niloticus*) post *Aeromonas hydrophila* infection.

Parameters	Control	Oregano 0.5 + sod 0.5	Oregano1 + sod 1	*p*. value
WBCs (x10³/mm³)	24.00$\:\pm\:$2.37^b^	51.83$\:\pm\:$4.87^a^	60.50$\:\pm\:$9.05^a^	0.0129
Heterophil (%)	23.00$\:\pm\:$0.57^a^	15.00$\:\pm\:$1.15^b^	17.67$\:\pm\:$2.60^ab^	0.0384
Lymphocyte (%)	65.67$\:\pm\:1.20$^b^	74.00$\:\pm\:$0.57^a^	72.33$\:\pm\:$2.96^ab^	0.0436
Monocyte (%)	8.33$\:\pm\:$0.88	8.66$\:\pm\:$0.88	8.00$\:\pm\:$0.57	0.8424
Eosinophil (%)	1.33$\:\pm\:$0.33	1.33$\:\pm\:$0.33	1.00$\:\pm\:$0	0.6297
Basophil (%)	1.66$\:\pm\:$0.33	1.00$\:\pm\:$0	1.00$\:\pm\:$0.57	0.4219
Heterophil (x10³/mm	5.51$\:\pm\:$0.51^b^	9.10$\:\pm\:$0.79^ab^	11.36$\:\pm\:$2.08^a^	0.0525
Lymphocyte (x10³/m	15.80$\:\pm\:$1.59^b^	38.30$\:\pm\:$3.48^a^	43.83$\:\pm\:$7.08^a^	0.0117
Monocyte (x10³/mm³)	1.97$\:\pm\:0.17$^b^	4.41$\:\pm\:$0.06^a^	4.84$\:\pm\:$0.84^a^	0.0136
Eosinophils(x10³/mm³)	0.33$\:\pm\:0.11$^b^	0.80$\:\pm\:$0.11^a^	0.60$\:\pm\:$0.09^ab^	0.0551
Basophil(x10³/mm³)	0.41$\:\pm\:$0.11^b^	0.51$\:\pm\:$0.04^ab^	0.74$\:\pm\:$0.05^a^	0.0500

Mean ± SEM where *n* = 3 as triplicate tanks. Values with different superscripts within a row significantly differ (*p* < 0.05).

**Table 7:** Effect of dietary level of oregano and sodium butyrate for 56 days on biochemical parameters of nile tilapia (*Oreochromis niloticus*) post *Aeromonas hydrophila* infection.

Parameters	Control	Oregano 0.5 + sod 0.5	Oregano1 + sod 1	*p*. value
ALT (U/l)	49.82$\:\pm\:$3.34^a^	33.83$\:\pm\:$4.46^ab^	30.67$\:\pm\:$4.95^b^	0.0412
AST (U/l)	46.75$\:\pm\:$6.68^a^	26.06$\:\pm\:$2.94^b^	30.83$\:\pm\:$3.05^ab^	0.0420
Total Protein (g/dl)	3.68$\:\pm\:$0.15^b^	4.64$\:\pm\:$0.27^a^	4.32$\:\pm\:$0.13^ab^	0.0327
Albumin (g/dl)	1.35$\:\pm\:$0.01	1.42$\:\pm\:$0.01	1.40$\:\pm\:$0.04	0.3771
Globulin (g/dl)	2.49$\:\pm\:0.16$	3.22$\:\pm\:$0.28	2.92$\:\pm\:0.08$	0.0979
Urea (mg/dl)	2.88$\:\pm\:$0.11	3.30$\:\pm\:$0.13	2.74$\:\pm\:0.33$	0.2514
Creatinine (mg/dl)	1.38$\:\pm\:$0.01^ab^	1.46$\:\pm\:$0.12^ab^	1.84$\:\pm\:$0.26^a^	0.1996

Mean ± SEM where *n* = 3 as triplicate tanks. Values with different superscripts within a row significantly differ (*p* < 0.05).

### Gene expression analysis

**Fig. 3:**
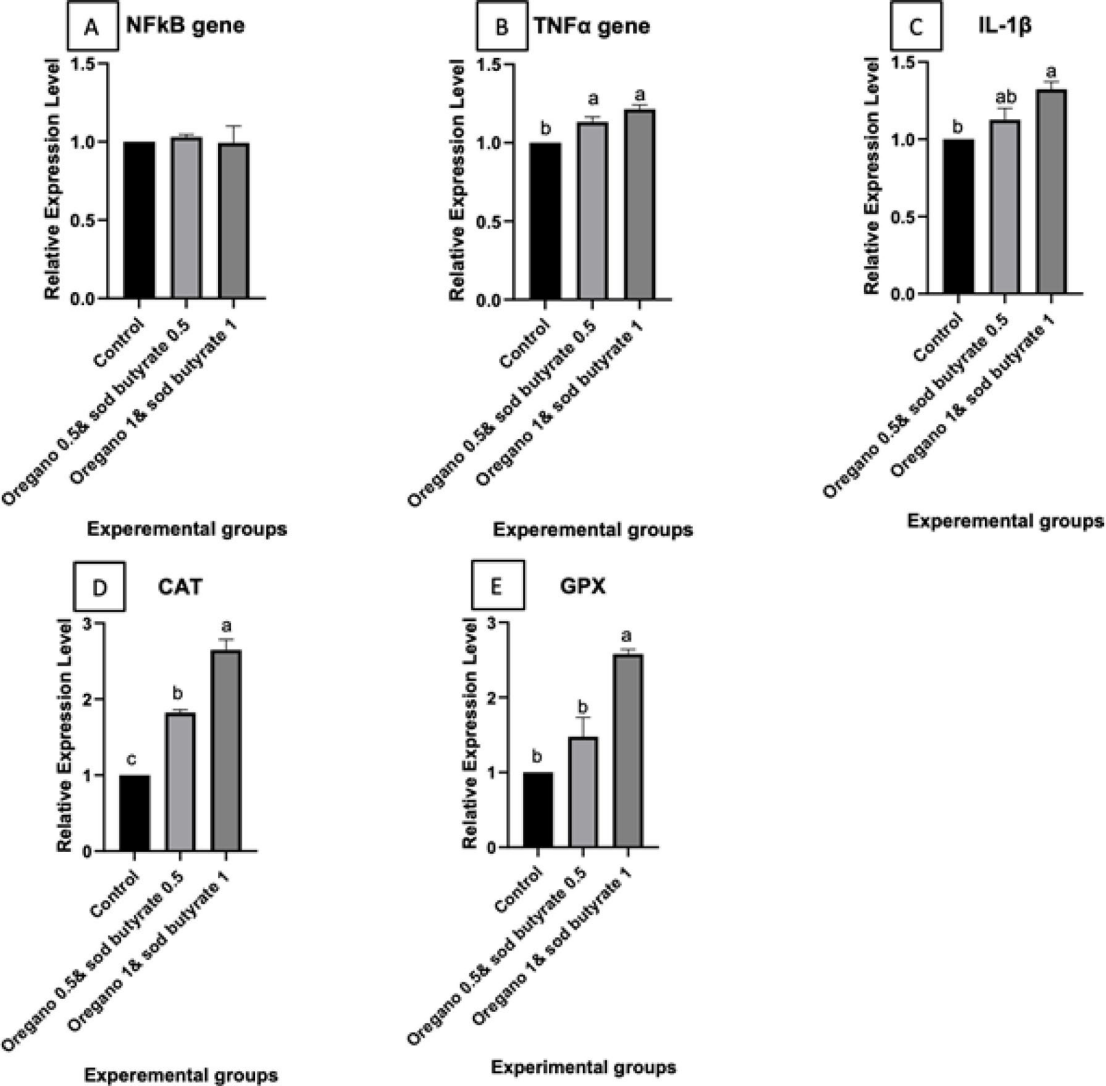
Differential expression of inflammation-related genes and antioxidant related genes in the spleen of Nile tilapia groups fed on oregano 0.5 and oregano 1.(before *A. hydrophila* infection) (**A**) Nf-κb: Nuclear factor kappa B, (**B**) TNF-α: Tumor necrosis factor alpha, (**C**) IL-1β: Interleukin 1β, (**D**) CAT: Catalase, (**E**), GPx: Glutathione peroxidase. Columns with different superscript letters in the exact figure same figure are significantly different (*p* ≤ 0.05). Are significantly different (*p* ≤ 0.05).

**Fig. 4:**
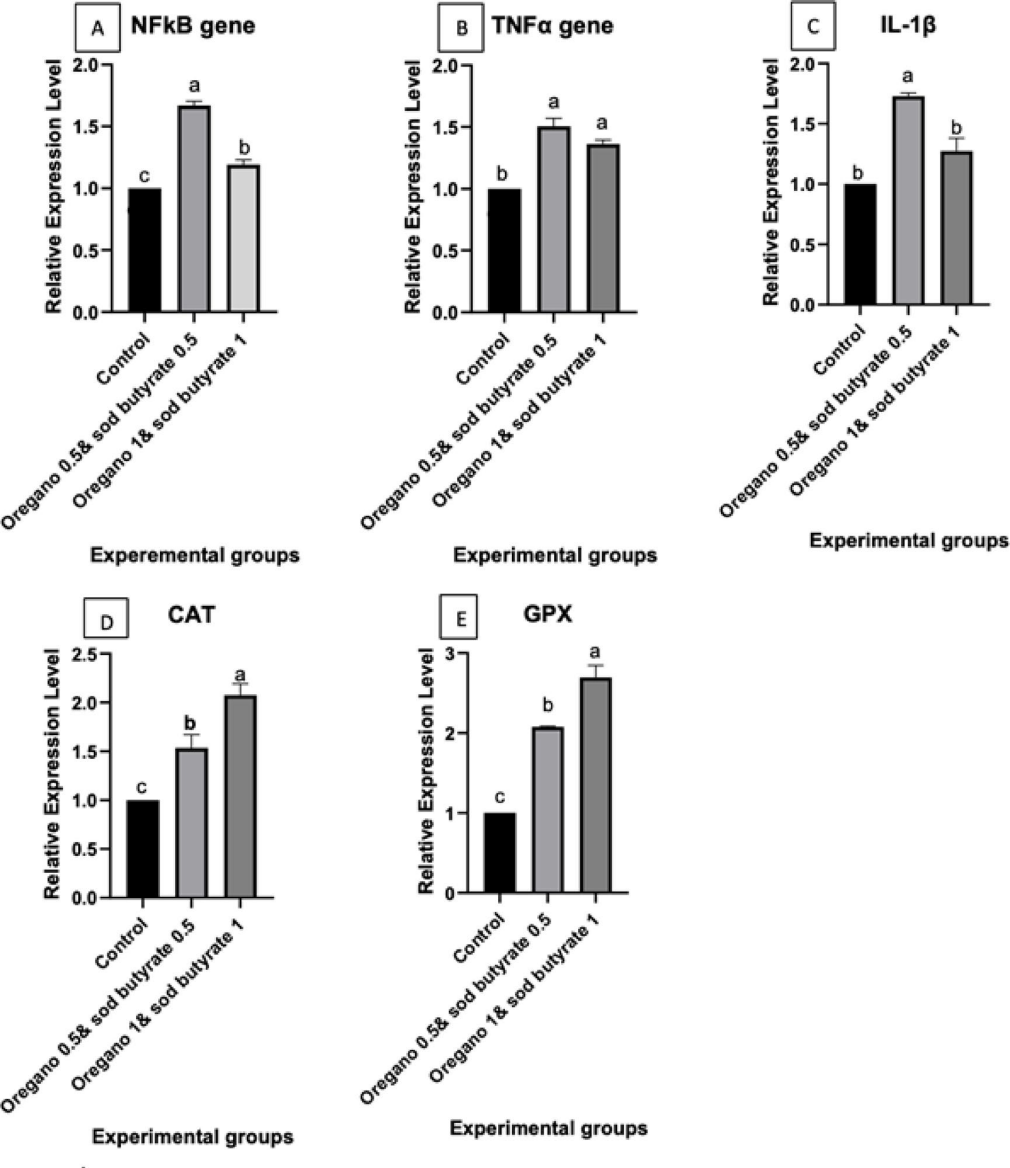
Differential expression of inflammation-related genes and antioxidant-related genes in the spleen of Nile tilapia groups fed on oregano 0.5 & sod butyrate 0.5 and oregano 1& sod butyrate 1.(After *A. hydrophila* infection) (**A**) Nf-κb: Nuclear factor kappa B, (**B**) TNF-α: Tumor necrosis factor alpha, (**C**) IL-1β: Interleukin 1β, (**D**) CAT: Catalase, (**E**), GPx: Glutathione peroxidase. Columns with different superscript letters in the exact figure same figure are significantly different (*p* ≤ 0.05).

The results demonstrate the differential expression of inflammation-related genes (NF-κB, TNF-α, IL-1β) and antioxidant-related genes (CAT, GPX) in the spleen of Nile tilapia-fed diets supplemented with oregano (0.5% and 1%) or combinations of oregano and sodium butyrate (0.5% & 1%), both before and after *Aeromonas hydrophila* infection. Before the infection, TNF-α and IL-1β expression significantly increased in groups fed oregano-supplemented diets compared to the control, whereas NF-κB expression levels remained unaffected. Antioxidant-related genes (CAT and GPX) also showed significantly elevated expression in the oregano-supplemented groups, particularly at the higher dose (1%), compared to the control. Figure 3. After *Aeromonas hydrophila* infection, *TNF-α* and *IL-1β* expressions were further upregulated in groups supplemented with oregano and sodium butyrate compared to the control. In contrast, NF-κB expression levels showed minimal variation. Additionally, CAT and GPX expression levels were markedly elevated in the oregano and sodium butyrate-supplemented groups after infection, with the highest expression observed at the 1% oregano + 1% sodium butyrate dose. These findings highlight that supplementation with oregano and sodium butyrate positively modulates immune and antioxidant gene expression, enhancing Nile tilapia’s immune and antioxidant responses, particularly following infection. Columns with different superscript letters indicate significant differences (*p* ≤ 0.05), Figure. 4.

### Morphometry of intestinal villi

**Fig. 5:**
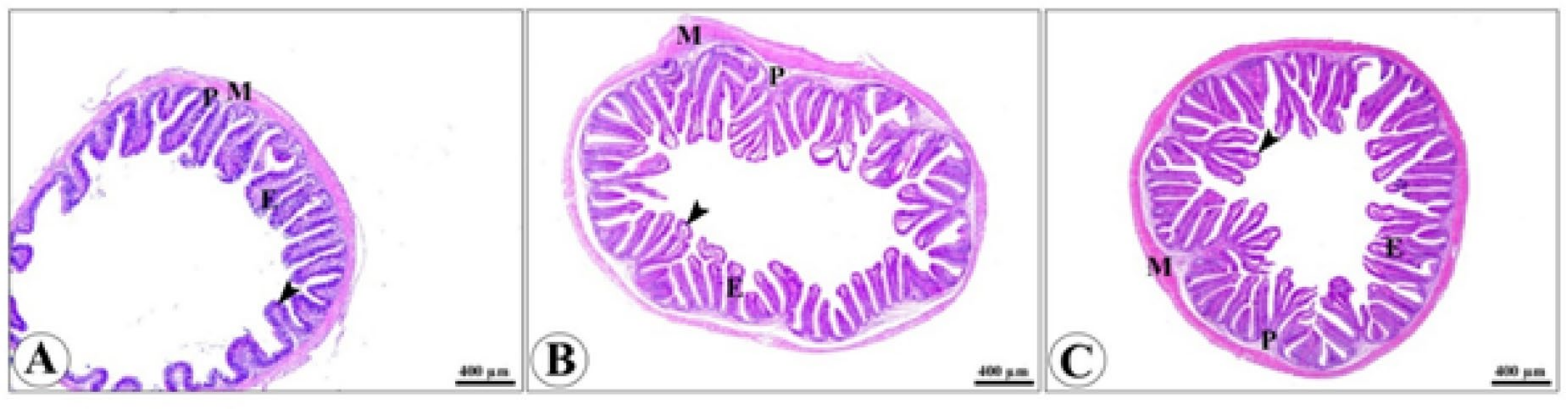
H&E-stained photomicrograph of the anterior part of the intestine of O. niloticus of control (**A**), oregano 0.5 & sod butyrate 0.5 (**B**) and oregano 1& sod butyrate 1 (**C**) showing intestinal villi (arrowheads), lamina epithelialis (E), lamina propria (P) and lamina muscularis (M).

The microscopic structure of the intestinal wall of fish is formed of tunica mucosa, composed of lamina epithelial, which contains numerous intestinal villi lined by simple columnar epithelium with goblet cells resting on lamina propria submucosa, followed by tunica muscularis and bounded externally by tunica serosa. The density, branching, and length of intestinal villi were more prominent in the treated groups when compared with the control non-treated group Figure. 5. The data morphometric analysis of the anterior part of the intestine is displayed in Table 8.

**Table 8:** Intestinal histopathological injury score in *Oreochromis niloticus* (anterior intestine) following 56-day feeding and post-*Aeromonas hydrophila* challenge.

	Control	Oregano 0.5 + sod 0.5	Oregano1 + sod 1	*p*. value
Villi length (µm)	387.7 ± 10.67^b^	518.9 ± 20.71^a^	582.7 ± 36.91^a^	0.0046
Villi width (µm)	148.6 ± 16.42	114.9 ± 7.920	120.4 ± 6.571	0.1142
Crypt depth (µm)	69.97 ± 3.321	69.08 ± 4.684	75.19 ± 3.741	0.5174
Villi length/crypt depth	5.487 ± 0.255^b^	8.076 ± 0.476^a^	8.282 ± 0.661^a^	0.0028

Mean ± SEM where *n* = 3 as triplicate tanks. Values with different superscripts within a row significantly differ (*p* < 0.05).

## Discussion

The findings of this study demonstrate that dietary supplementation with oregano essential oil and sodium butyrate significantly improved the growth performance, feed utilization, biochemical profile, antioxidant status, immune response, and gene expression in Nile tilapia, both under normal conditions and following challenge with *Aeromonas hydrophila*. These improvements highlight the synergistic effects of both additives in enhancing systemic resilience and physiological homeostasis in aquaculture species. When oregano and sodium butyrate were incorporated into the diets, Nile tilapia exhibited marked enhancements in overall health and performance. This was evident in the significantly higher final body weight and weight gain observed in the supplemented groups compared to the control. Such results confirm the positive impact of these feed additives on growth promotion and underline their potential as functional dietary components for boosting fish productivity and disease resistance. The improvements in growth performance are likely due to enhanced feed utilization and gut health. Oregano essential oil contains bioactive compounds such as carvacrol and thymol, which have well-documented antimicrobial properties that help reduce harmful gut bacteria, creating a more favorable environment for nutrient absorption^25^. Additionally, sodium butyrate, as a short-chain fatty acid, serves as a key energy source for intestinal epithelial cells and promotes intestinal barrier function and mucosal integrity, thereby improving nutrient uptake and feed conversion efficiency^47^.

Among the groups tested, 1% oregano and 1% sodium butyrate had the most significant improvement, while 0.5% oregano and 0.5% sodium butyrate came in second. The antibacterial and gastrointestinal health-enhancing actions of oregano and sodium butyrate may be responsible for this growth spurt^22,48^. The FCR and SGR were also adjusted, enhancing feed efficiency and nutrient absorption^49^. Hematological parameters were significantly improved, including RBCs, Hb, and PCV. The supplemented groups exhibited higher values, indicating improved oxygen-carrying capacity and overall health. These changes were also linked to enhanced immune function, as better blood parameters can improve tissue oxygenation and resistance to infections^50^.

The supplementation positively affected biochemical markers, such as ALT, AST, total protein, albumin, globulin, urea, and creatinine. Lower levels of ALT and AST suggested improved liver function, while higher concentrations of total protein, albumin, and globulin reflected better protein metabolism and immune response^51^. From a biochemical perspective, the observed reductions in ALT and AST levels in supplemented groups indicate better liver function and reduced hepatocellular damage, likely mediated by both additives’ antioxidant and membrane-stabilizing effects. The increase in total protein and globulin levels suggests enhanced protein synthesis and a more robust immune response, possibly associated with improved liver function and activation of the humoral immune system. Reduced urea and creatinine levels indicate better kidney function and waste nitrogen metabolism^52^.

The supplementation significantly enhanced white blood cell (WBC) count, including monocytes, and improved phagocytic activity, indicating a robust immune response^53^. The antioxidative enzyme activities of SOD, CAT, and GPX were also elevated in the supplemented groups, pointing to better oxidative stress defense. The antioxidant defense system was markedly enhanced by the supplementation, as evidenced by elevated activities of SOD, CAT, and GPX enzymes, alongside a reduction in MDA levels. These findings suggest a decrease in lipid peroxidation and oxidative stress. Mechanistically, the antioxidant effects of oregano oil may be attributed to the activation of the Nrf2 signaling pathway by carvacrol and thymol, which upregulate the expression of genes encoding antioxidant enzymes^54^. Similarly, sodium butyrate has been shown to act as a histone deacetylase (HDAC) inhibitor, modifying chromatin structure and promoting the transcription of antioxidant and protective genes^21^. Lower MDA levels, a marker of oxidative damage, further suggested that the additives mitigated oxidative stress, a significant factor in aquaculture’s poor growth and immune suppression^55^.

Following exposure to *Aeromonas hydrophila*, the supplemented groups showed enhanced immune parameters, including higher WBC count, phagocytic activity, and lysozyme activity, demonstrating improved infection resistance^56^. Regarding gene expression, both oregano and sodium butyrate treatments increased TNF-α expression, suggesting immune system priming. The highest dose group (1% oregano and 1% sodium butyrate) showed elevated IL-1β expression, indicating an enhanced inflammatory response. Antioxidant gene expression was also higher in this group, suggesting improved oxidative stress defense. After infection, NF-κB expression was more pronounced in the lower dose group (0.5% oregano and sodium butyrate), indicating stronger immune activation. At the same time, *TNF-α* levels remained elevated across both supplemented groups. Gene expression analysis further supports the functional roles of these additives. The upregulation of *GPX* and *CAT* genes aligns with the observed increase in enzymatic activity, indicating transcriptional regulation. Moreover, the immune-related genes *IL-1β* and *TNF-α* were significantly upregulated in supplemented groups, both before and after infection, suggesting enhanced immune readiness and responsiveness. Butyrate’s role in modulating immune gene expression is well established and is believed to occur through epigenetic mechanisms and inhibition of inflammatory cytokines^57^. Likewise, the pro-inflammatory and immunostimulatory effects of oregano oil are mediated through toll-like receptor activation and cytokine signaling^58^.

Post-infection data showed that fish receiving oregano and butyrate supplementation maintained better biochemical stability and stronger antioxidant and immune responses, indicating increased resistance to *A. hydrophila*. This may be attributed to both local effects on gut immunity and systemic modulation of immune pathways, further supported by the histopathological improvements observed in intestinal villi structure. The combined supplementation of oregano essential oil and sodium butyrate offers multifaceted benefits to Nile tilapia by improving gut health, antioxidant capacity, immune function, and resistance to bacterial infection. These effects are mediated through a complex interplay of antimicrobial action, epigenetic modulation, and upregulation of protective gene pathways, highlighting their potential as sustainable alternatives to antibiotics in aquaculture. The study concluded that oregano and sodium butyrate supplementation enhanced growth performance and improved immune function and antioxidative capacity, particularly under stress conditions. These data are coherent with earlier research on the immunological and antioxidative impacts of oregano and sodium butyrate in fish (Júnior et al., 2017; Mai et al., 2018). The additives’ beneficial effects on growth, health, and immunity underscore their potential as functional ingredients in aquaculture diets.

Regarding safety, sodium butyrate is classified as ‘generally recognized as safe’ (GRAS) and is approved for animal feed across various regions. It is metabolized in the gastrointestinal tract and does not accumulate in fish tissues, minimizing food safety concerns. However, we recognize the need for long-term studies under commercial farming conditions to assess its chronic effects and environmental interactions further^18^.

We acknowledge that the current design did not independently evaluate each additive or explore multiple ratios to determine synergy or antagonism. Our goal in this preliminary investigation was to assess the biological plausibility of equal-dose combination therapy as a simplified dietary strategy. Future research should explore more complex dose–response and interaction models to validate and optimize these findings. The additives were used continuously during the experimental period to evaluate their full physiological impact under normal and pathogen-challenged conditions. However, we acknowledge that strategic or periodic administration in a real-world aquaculture setting (e.g., during early grow-out stages or disease-prone seasons) may be more cost-effective and sustainable. Based on our results, this feeding strategy may be suitable for use in either short-term immune priming or continuous application during critical periods, though further cost–benefit and long-term safety studies are recommended to define optimal usage schedules.

## Conclusion

In conclusion, dietary supplementation with oregano essential oil and sodium butyrate had a significant positive impact on the growth performance, hematological and biochemical parameters, antioxidant capacity, immune response, and disease resistance of Nile tilapia (*Oreochromis niloticus*), particularly following *Aeromonas hydrophila* infection. The most pronounced effects were observed in the group receiving the combined supplementation of 1% oregano essential oil and 1% sodium butyrate, which resulted in enhanced growth metrics, improved liver and kidney function markers, elevated antioxidant enzyme activity, upregulated expression of immune- and antioxidant-related genes, and superior post-infection survival. These findings support the potential use of these natural feed additives as effective alternatives to antibiotics in aquaculture. Further long-term studies under commercial farming conditions are recommended to validate these outcomes and determine optimal dosing strategies for sustainable tilapia production. Future investigations should assess these additives’ long-term application, intermittent dosing schedules, and economic feasibility in commercial-scale production systems.

## Supplementary Information

Below is the link to the electronic supplementary material.

Supplementary Material 1

## Data Availability

The authors confirm that all data supporting the findings of this study are presented within the article. Raw data can be made available upon reasonable request from the corresponding author.
