# Supplementary material for: Dietary oregano essential oil and sodium butyrate enhance growth, immunity, and gene expression in nile tilapia post-Aeromonas hydrophila infection
Source: Sci Rep. 2025 Nov 4;15:38496. doi: 10.1038/s41598-025-22439-8 (PMC12586456; doi:10.1038/s41598-025-22439-8)
Supplement: Supplementary file 1 — Supplementary Material 1 [file 41598_2025_22439_MOESM1_ESM.pdf]

Table S1. Experimental diet and composition of nutrients of Nile tilapia:

Feedstuff	T1	T2	T3
Fish meal (72%)	95	95	95
Corn gluten meal	100	100	100
Soybean meal (48% cp)	233	233	233
Corn grains, yellow	230	230	230
Wheat bran	134	134	134
Wheat middling	155	154	153
Fish oil	35	35	35
Monocalcium phosphate	5	5	5
Limestone	3.8	3.8	3.8
DL- methionine	1.2	1.2	1.2
Premix[1]	3	3	3
CMC[2]	5	5	5
Oregano oil + sodium butyrate	-	0.5+0.5	1+1
Nutrients %			
Crude protein	29.47	29.45	29.43
Starch	25	25	25
Lipids	7.05	7.05	7.04
DE (kcal/kg diet)	3126.8	3123.3	3120.4
Lysine	1.43	1.43	1.43
Methionine	0.75	0.75	0.75
Calcium	0.7	0.7	0.7
Available phosphorus	0.45	0.45	0.45

[1]premix; contained 1000 mg of copper, 1000 mg of iodine, 100 mg of selenium, 1 mg of cobalt, 100,000 mg of iron, 10,000 mg of manganese, 30,000 mg of zinc, 200,000 IU of vitamin A, 10,000 mg of vitamin E, and 2000 IU of vitamin D3.

1000 mg folic acid, 1000 mg B1 and B2; 4000 mg B6 and 4 mg B12; 20,000 mg niacin; 20 mg biotin; 10,000 mg pantothenic acid; calcium carbonates up to 1000 gm per kilogram

[2]CMC binder; (carboxy methyl cellulose)

Table S2: Primers used for qRT-PCR analysis.

Gene	Primer	Reference	Accession number
β-actin	F: CAGCAAGCAGGAGTACGATGAG<br>R: TGTGTGGTGTGTGGTTGTTTTG	(El-Kassas et al., 2020)	KJ126772
eef1a1l3	F: TCAACGCTCAGGTCATCATC<br>R: ACGGTCGATCTTCTCAACCA	(Con et al., 2019)	XM_019363060.2
nfkb1	F: GAACATCAGACCGACGACCA<br>R: TCTCCGCCAGTTTCTTCCA	(Abdo et al 2022)	XM_019363515.2
Il-1β	F: TCAGTTCACCAGCAGGGATG<br>R: GACAGATAGAGGTTTGTGCC	(Abdo et al., 2022)	KF747686.1
TNFα	F: AAGCCAAGGCAGCCATCCAT<br>R: TTGACCATTCCTCCACTCCAGA	(Limbu et al., 2018)	AY428948.1
CAT	F: CCCAGCTCTTCATCCAGAAAC<br>R: GCCTCCGCATTGTACTTCTT	(Abdo et al., 2021)	JF801726.1
GPX	F: CCAAGAGAACTGCAAGAACGA<br>R: CAGGACACGTCATTCCTACAC	(El-Kassas et al., 2022)	DQ355022.1

*Internal reference genes (β-actin) and elongation factor 1 alpha (eef1a1l3), nuclear factor kappa B (**nfkb1**), interleukin 1β (IL-1β), tumor necrosis factor alpha (TNFα), catalase (CAT), glutathione peroxidase (GPx),*
